# Supplementary material for: Amyloid-β Pathology-Specific Cytokine Secretion Suppresses Neuronal Mitochondrial Metabolism
Source: Cell Mol Bioeng. 2023 Sep 11;16(4):405–21. doi: 10.1007/s12195-023-00782-y (PMC10550897; doi:10.1007/s12195-023-00782-y)
Supplement: Supplementary file 1 — Supplementary file1 (DOCX 21 KB) [file 12195_2023_782_MOESM1_ESM.docx]

Supplementary Table 1: Significantly differentially expressed genes of cytokine-treated neurons compared to vehicle-treated neurons

| \| **Gene** \| **log2FC** \| **P-value** \| \| --- \| --- \| --- \| \| A2m \| -0.91 \| 0.043 \| \| Acaa2 \| -0.8 \| 0.003 \| \| Acadl \| 0.2 \| 0.001 \| \| Acox1 \| -0.44 \| 0.004 \| \| Ak1 \| -0.19 \| 0.028 \| \| Ak3 \| -0.37 \| 0.040 \| \| Aldh2 \| -0.43 \| 0.015 \| \| Apoe \| -0.52 \| 0.030 \| \| Ar \| 0.64 \| 0.043 \| \| Atf4 \| 0.3 \| 0.021 \| \| Atf7ip \| 0.37 \| 0.004 \| \| Atp5d \| -0.26 \| 0.010 \| \| Birc3 \| 1.01 \| 0.021 \| \| Brip1 \| 1.03 \| 0.016 \| \| Bub1 \| -0.47 \| 0.007 \| \| Cacng2 \| -0.43 \| 0.026 \| \| Cat \| -0.23 \| 0.014 \| \| Ccl19 \| 1.56 \| 0.001 \| \| Ccl2 \| 3.44 \| 0.001 \| \| Ccl4 \| -2.75 \| 0.011 \| \| Ccl5 \| 5.18 \| 0.001 \| \| Ccna2 \| -0.68 \| 0.009 \| \| Cd14 \| -2.32 \| 0.002 \| \| Cd274 \| 4.94 \| 0.001 \| \| Cd36 \| 4.36 \| 0.000 \| \| Cdc20 \| -0.39 \| 0.048 \| \| Cdk9 \| 0.09 \| 0.006 \| \| Cenpa \| -0.56 \| 0.015 \| \| Ctsl \| -0.62 \| 0.035 \| \| Cxcl9 \| 5.13 \| 0.004 \| \| Cyp1b1 \| -0.17 \| 0.033 \| \| Dck \| 0.3 \| 0.040 \| \| Dguok \| 0.34 \| 0.020 \| \| Epc1 \| 0.33 \| 0.006 \| \| Fcf1 \| 0.35 \| 0.031 \| \| Fcgr4 \| 2.71 \| 0.015 \| \| Fcrls \| -3.06 \| 0.013 \| \| Fdx1 \| 0.39 \| 0.012 \| \| Fgf1 \| -1.05 \| 0.002 \| \| Fnip1 \| 0.18 \| 0.031 \| \| Foxm1 \| -0.6 \| 0.005 \| \| Fpr1 \| 1.68 \| 0.011 \| \| Gad1 \| -0.52 \| 0.044 \| \| Gclc \| -0.26 \| 0.024 \| \| Glrx \| 0.71 \| 0.011 \| \| Glul \| -0.86 \| 0.016 \| \| Gmpr \| -0.69 \| 0.008 \| \| Gng12 \| 0.24 \| 0.021 \| \| Gpx1 \| 0.89 \| 0.006 \| \| Gusb \| -0.81 \| 0.042 \| \| H2-Aa \| 9.43 \| 0.001 \| \| H2-D1 \| 2.74 \| 0.019 \| | \| **Gene** \| **log2FC** \| **P-value** \| \| --- \| --- \| --- \| \| H2-DMa \| 4.32 \| 0.000 \| \| H2-Eb1 \| 7.61 \| 0.001 \| \| H2-M3 \| 2.13 \| 0.024 \| \| H2-T23 \| 4.42 \| 0.014 \| \| Hacd2 \| -0.5 \| 0.017 \| \| Hadh \| -0.18 \| 0.004 \| \| Hdc \| 1.75 \| 0.002 \| \| Hk2 \| 0.72 \| 0.011 \| \| Hk3 \| 2.25 \| 0.014 \| \| Hsd11b1 \| -1.16 \| 0.032 \| \| Hspe1 \| 0.26 \| 0.010 \| \| Idh3g \| -0.23 \| 0.048 \| \| Idnk \| 1.3 \| 0.000 \| \| Irf1 \| 5.15 \| 0.000 \| \| Itch \| 0.18 \| 0.030 \| \| Itga1 \| -0.64 \| 0.021 \| \| Jak2 \| 0.97 \| 0.000 \| \| Keap1 \| 0.51 \| 0.002 \| \| Kif2c \| -0.46 \| 0.028 \| \| Klrk1 \| 5.22 \| 0.002 \| \| Kmt2a \| 0.41 \| 0.050 \| \| Kyat1 \| -0.39 \| 0.014 \| \| Lamc1 \| 0.55 \| 0.035 \| \| Lamtor2 \| 0.13 \| 0.001 \| \| Lck \| 1.24 \| 0.020 \| \| Ldha \| -0.41 \| 0.036 \| \| Ldhb \| -0.37 \| 0.000 \| \| Map1lc3b \| -0.37 \| 0.002 \| \| Map2k2 \| 0.21 \| 0.010 \| \| Mki67 \| -0.86 \| 0.005 \| \| Msrb2 \| -0.6 \| 0.009 \| \| Mtf1 \| -0.16 \| 0.032 \| \| Myb \| -0.65 \| 0.007 \| \| Myd88 \| 1.2 \| 0.007 \| \| Nadk \| 0.35 \| 0.023 \| \| Ncoa2 \| 0.33 \| 0.021 \| \| Ndufa1 \| -0.15 \| 0.005 \| \| Ndufa4 \| -0.37 \| 0.032 \| \| Nfs1 \| -0.13 \| 0.042 \| \| Nme2 \| 0.21 \| 0.020 \| \| Npm1 \| 0.2 \| 0.010 \| \| Nqo1 \| -1.05 \| 0.008 \| \| Nras \| 0.31 \| 0.027 \| \| Pclaf \| -0.5 \| 0.029 \| \| Pgk1 \| -0.38 \| 0.017 \| \| Pik3cb \| -0.26 \| 0.040 \| \| Pik3r3 \| 0.54 \| 0.008 \| \| Plk1 \| -0.87 \| 0.005 \| \| Pole \| -0.58 \| 0.037 \| \| Prdx5 \| 0.38 \| 0.001 \| \| Prim2 \| -0.22 \| 0.016 \| \| Prkag1 \| -0.22 \| 0.009 \| | \| **Gene** \| **log2FC** \| **P-value** \| \| --- \| --- \| --- \| \| Psat1 \| -0.34 \| 0.030 \| \| Psma3 \| 0.49 \| 0.001 \| \| Psma7 \| 0.72 \| 0.003 \| \| Psmb10 \| 3.27 \| 0.004 \| \| Psmb3 \| 0.34 \| 0.001 \| \| Psmc1 \| 0.14 \| 0.014 \| \| Psmd13 \| 0.15 \| 0.046 \| \| Psme2 \| 2.2 \| 0.002 \| \| Psph \| -0.16 \| 0.015 \| \| Ptger4 \| -0.54 \| 0.022 \| \| Ptges \| 1.22 \| 0.001 \| \| Rgn \| 0.81 \| 0.046 \| \| Rplp0 \| 0.28 \| 0.027 \| \| Rps6kb1 \| 0.3 \| 0.014 \| \| Rrm1 \| -0.26 \| 0.006 \| \| Rrm2 \| -0.69 \| 0.006 \| \| Scd1 \| -0.44 \| 0.001 \| \| Sdhb \| 0.49 \| 0.006 \| \| Sdhc \| -0.05 \| 0.012 \| \| Sec13 \| 0.12 \| 0.035 \| \| Slc16a1 \| 0.19 \| 0.027 \| \| Slc16a2 \| -0.46 \| 0.001 \| \| Slc16a3 \| -0.5 \| 0.001 \| \| Slc1a5 \| -1.14 \| 0.002 \| \| Slc25a1 \| 0.1 \| 0.010 \| \| Slc7a5 \| -0.36 \| 0.007 \| \| Smad4 \| 0.17 \| 0.022 \| \| Sox2 \| 0.29 \| 0.011 \| \| Sqstm1 \| 0.31 \| 0.001 \| \| Stat1 \| 4.05 \| 0.006 \| \| Stat3 \| 0.76 \| 0.001 \| \| Stat5a \| 0.46 \| 0.020 \| \| Tbk1 \| 0.37 \| 0.020 \| \| Tkt \| -0.2 \| 0.022 \| \| Tlr1 \| 0.76 \| 0.023 \| \| Tnf \| 1.76 \| 0.023 \| \| Tpr \| 0.22 \| 0.025 \| \| Traf1 \| 1.02 \| 0.001 \| \| Traf6 \| 0.17 \| 0.023 \| \| Trf \| -0.82 \| 0.045 \| \| Trp53 \| 0.4 \| 0.016 \| \| Trp63 \| -0.83 \| 0.017 \| \| Txn1 \| 0.81 \| 0.002 \| \| Ubb \| 0.38 \| 0.003 \| \| Upp1 \| 0.79 \| 0.008 \| \| Uqcr11 \| -0.32 \| 0.045 \| \| Usp39 \| 0.35 \| 0.011 \| \| Usp8 \| 0.39 \| 0.006 \| \| Washc4 \| 0.4 \| 0.020 \| \| Wrn \| 1.06 \| 0.001 \| \| Zfp65 \| 0.31 \| 0.005 \| \| Zfp869 \| 0.44 \| 0.006 \| |
| --- | --- | --- | --- | --- | --- | --- | --- | --- | --- | --- | --- | --- | --- | --- | --- | --- | --- | --- | --- | --- | --- | --- | --- | --- | --- | --- | --- | --- | --- | --- | --- | --- | --- | --- | --- | --- | --- | --- | --- | --- | --- | --- | --- | --- | --- | --- | --- | --- | --- | --- | --- | --- | --- | --- | --- | --- | --- | --- | --- | --- | --- | --- | --- | --- | --- | --- | --- | --- | --- | --- | --- | --- | --- | --- | --- | --- | --- | --- | --- | --- | --- | --- | --- | --- | --- | --- | --- | --- | --- | --- | --- | --- | --- | --- | --- | --- | --- | --- | --- | --- | --- | --- | --- | --- | --- | --- | --- | --- | --- | --- | --- | --- | --- | --- | --- | --- | --- | --- | --- | --- | --- | --- | --- | --- | --- | --- | --- | --- | --- | --- | --- | --- | --- | --- | --- | --- | --- | --- | --- | --- | --- | --- | --- | --- | --- | --- | --- | --- | --- | --- | --- | --- | --- | --- | --- | --- | --- | --- | --- | --- | --- | --- | --- | --- | --- | --- | --- | --- | --- | --- | --- | --- | --- | --- | --- | --- | --- | --- | --- | --- | --- | --- | --- | --- | --- | --- | --- | --- | --- | --- | --- | --- | --- | --- | --- | --- | --- | --- | --- | --- | --- | --- | --- | --- | --- | --- | --- | --- | --- | --- | --- | --- | --- | --- | --- | --- | --- | --- | --- | --- | --- | --- | --- | --- | --- | --- | --- | --- | --- | --- | --- | --- | --- | --- | --- | --- | --- | --- | --- | --- | --- | --- | --- | --- | --- | --- | --- | --- | --- | --- | --- | --- | --- | --- | --- | --- | --- | --- | --- | --- | --- | --- | --- | --- | --- | --- | --- | --- | --- | --- | --- | --- | --- | --- | --- | --- | --- | --- | --- | --- | --- | --- | --- | --- | --- | --- | --- | --- | --- | --- | --- | --- | --- | --- | --- | --- | --- | --- | --- | --- | --- | --- | --- | --- | --- | --- | --- | --- | --- | --- | --- | --- | --- | --- | --- | --- | --- | --- | --- | --- | --- | --- | --- | --- | --- | --- | --- | --- | --- | --- | --- | --- | --- | --- | --- | --- | --- | --- | --- | --- | --- | --- | --- | --- | --- | --- | --- | --- | --- | --- | --- | --- | --- | --- | --- | --- | --- | --- | --- | --- | --- | --- | --- | --- | --- | --- | --- | --- | --- | --- | --- | --- | --- | --- | --- | --- | --- | --- | --- | --- | --- | --- | --- | --- | --- | --- | --- | --- | --- | --- | --- | --- | --- | --- | --- | --- | --- | --- | --- | --- | --- | --- | --- | --- | --- | --- | --- | --- | --- | --- | --- | --- | --- | --- | --- | --- | --- | --- | --- | --- | --- | --- | --- | --- | --- | --- | --- | --- | --- | --- | --- | --- | --- | --- | --- | --- | --- | --- | --- | --- | --- | --- | --- | --- | --- | --- | --- | --- | --- | --- | --- | --- | --- | --- | --- | --- | --- | --- | --- | --- | --- | --- | --- | --- | --- | --- | --- | --- | --- | --- | --- | --- | --- | --- | --- | --- | --- | --- | --- |

Supplementary Table 2: Significantly differentially expressed genes of cytokine-treated astrocytes compared to vehicle-treated astrocytes

| \| \| **Gene** \| **log2FC** \| **P-value** \| \| --- \| --- \| --- \| \| Ada \| 0.73 \| 0.049 \| \| Akt3 \| 0.48 \| 0.002 \| \| Cab39 \| -0.22 \| 0.025 \| \| Ccl2 \| 2.88 \| 0.037 \| \| Ccl5 \| 3.55 \| 0.016 \| \| Cd180 \| 0.7 \| 0.020 \| \| Cd274 \| 5.36 \| 0.000 \| \| Ctss \| 1.63 \| 0.003 \| \| Cxcl9 \| 3.21 \| 0.017 \| \| Cybb \| 1.2 \| 0.002 \| \| Dnajc14 \| -0.15 \| 0.043 \| \| Fcgr4 \| 2.02 \| 0.001 \| \| Fgf1 \| -0.85 \| 0.003 \| \| Gns \| 0.23 \| 0.040 \| \| Gsk3b \| -0.29 \| 0.020 \| \| H2-Aa \| 8.65 \| 0.001 \| \| H2-D1 \| 5.79 \| 0.000 \| \| H2-DMa \| 5.32 \| 0.000 \| \| H2-Eb1 \| 6.87 \| 0.001 \| \| H2-M3 \| 4.61 \| 0.000 \| \| H2-T23 \| 4 \| 0.003 \| \| Hexa \| 0.27 \| 0.003 \| \| Hk3 \| 2.07 \| 0.008 \| \| Hspa2 \| 0.52 \| 0.011 \| \| Hspa4 \| -0.39 \| 0.001 \| \| Idh2 \| -0.49 \| 0.036 \| \| Idnk \| 1.31 \| 0.007 \| \| Irf1 \| 4.39 \| 0.001 \| \| Itgb2 \| 0.91 \| 0.041 \| \| Ldha \| -0.46 \| 0.025 \| \| Ly86 \| 0.54 \| 0.045 \| \| Map2k1 \| 0.1 \| 0.028 \| \| Mlst8 \| -0.39 \| 0.026 \| \| Mycn \| -0.5 \| 0.021 \| \| Ndufa3 \| 0.17 \| 0.028 \| \| Pik3r1 \| -0.4 \| 0.035 \| \| Prkcg \| -0.63 \| 0.032 \| \| Psma3 \| 0.46 \| 0.008 \| \| Psma7 \| 0.55 \| 0.009 \| \|  \|  \| \| --- \| --- \| --- \| --- \| --- \| --- \| --- \| --- \| --- \| --- \| --- \| --- \| --- \| --- \| --- \| --- \| --- \| --- \| --- \| --- \| --- \| --- \| --- \| --- \| --- \| --- \| --- \| --- \| --- \| --- \| --- \| --- \| --- \| --- \| --- \| --- \| --- \| --- \| --- \| --- \| --- \| --- \| --- \| --- \| --- \| --- \| --- \| --- \| --- \| --- \| --- \| --- \| --- \| --- \| --- \| --- \| --- \| --- \| --- \| --- \| --- \| --- \| --- \| --- \| --- \| --- \| --- \| --- \| --- \| --- \| --- \| --- \| --- \| --- \| --- \| --- \| --- \| --- \| --- \| --- \| --- \| --- \| --- \| --- \| --- \| --- \| --- \| --- \| --- \| --- \| --- \| --- \| --- \| --- \| --- \| --- \| --- \| --- \| --- \| --- \| --- \| --- \| --- \| --- \| --- \| --- \| --- \| --- \| --- \| --- \| --- \| --- \| --- \| --- \| --- \| --- \| --- \| --- \| --- \| --- \| --- \| --- \| --- \| | \| **Gene** \| **log2FC** \| **P-value** \| \| --- \| --- \| --- \| \| Psmb10 \| 3.73 \| 0.000 \| \| Psmb3 \| 0.35 \| 0.006 \| \| Psmc1 \| 0.17 \| 0.013 \| \| Psmd13 \| 0.16 \| 0.042 \| \| Psme2 \| 2.17 \| 0.000 \| \| Ptk2 \| -0.25 \| 0.015 \| \| Pycr1 \| 0.59 \| 0.021 \| \| Rbks \| -0.34 \| 0.025 \| \| Sdha \| -0.25 \| 0.050 \| \| Slc25a1 \| 0.23 \| 0.014 \| \| Sqstm1 \| 0.29 \| 0.044 \| \| Stat1 \| 5.3 \| 0.000 \| \| Tyms \| -0.44 \| 0.033 \| \| Washc4 \| 0.3 \| 0.000 \| \| Wrn \| 0.53 \| 0.048 \| \| Xdh \| 1.67 \| 0.006 \| \| Zfp457 \| -0.67 \| 0.001 \| |
| --- | --- | --- | --- | --- | --- | --- | --- | --- | --- | --- | --- | --- | --- | --- | --- | --- | --- | --- | --- | --- | --- | --- | --- | --- | --- | --- | --- | --- | --- | --- | --- | --- | --- | --- | --- | --- | --- | --- | --- | --- | --- | --- | --- | --- | --- | --- | --- | --- | --- | --- | --- | --- | --- | --- | --- | --- | --- | --- | --- | --- | --- | --- | --- | --- | --- | --- | --- | --- | --- | --- | --- | --- | --- | --- | --- | --- | --- | --- | --- | --- | --- | --- | --- | --- | --- | --- | --- | --- | --- | --- | --- | --- | --- | --- | --- | --- | --- | --- | --- | --- | --- | --- | --- | --- | --- | --- | --- | --- | --- | --- | --- | --- | --- | --- | --- | --- | --- | --- | --- | --- | --- | --- | --- | --- | --- | --- | --- | --- | --- | --- | --- | --- | --- | --- | --- | --- | --- | --- | --- | --- | --- | --- | --- | --- | --- | --- | --- | --- | --- | --- | --- | --- | --- | --- | --- | --- | --- | --- | --- | --- | --- | --- | --- | --- | --- | --- | --- | --- | --- | --- | --- | --- | --- | --- | --- | --- | --- | --- |
