# Supplementary material for: Amyloid-β Pathology-Specific Cytokine Secretion Suppresses Neuronal Mitochondrial Metabolism
Source: Cell Mol Bioeng. 2023 Sep 11;16(4):405–21. doi: 10.1007/s12195-023-00782-y (PMC10550897; doi:10.1007/s12195-023-00782-y)
Supplement: Supplementary file 2 — Supplementary file2 (PDF 318 KB) [file 12195_2023_782_MOESM2_ESM.pdf]

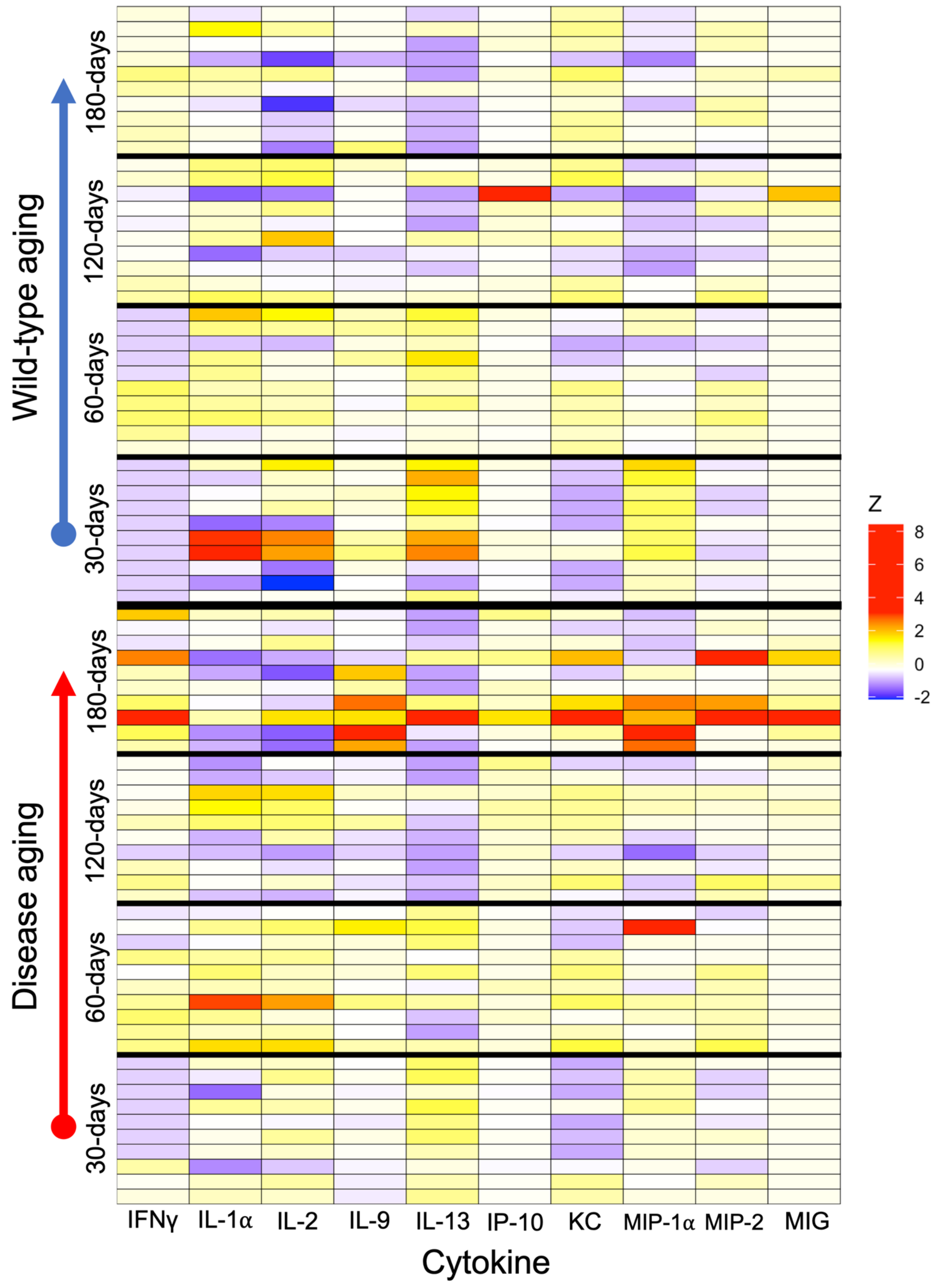

Supplementary Figure 1. Heat-map of Z-scored hippocampal cytokine concentrations. Normalized cytokine concentrations in individual animals from each genotype-age grouping. No individual cytokines emerge as unilaterally up- or down-regulated over the course of disease progression vs. normal aging.
